# The Role and Clinical Implications of the Retinoblastoma (RB)-E2F Pathway in Gastric Cancer

**DOI:** 10.3389/fonc.2021.655630

**Published:** 2021-05-31

**Authors:** Tianyi Wu, Lizhao Wu

**Affiliations:** Department of Pathophysiology, College of Basic Medical Sciences, China Medical University, Shenyang, China

**Keywords:** gastric cancer, RB-E2F pathway, pocket protein, E2F family, *Helicobacter pylori*, p53

## Abstract

Gastric cancer is the most common malignant tumor in the digestive tract, with very high morbidity and mortality in developing countries. The pathogenesis of gastric cancer is a complex biological process mediated by abnormal regulation of proto-oncogenes and tumor suppressor genes. Although there have been some in-depth studies on gastric cancer at the molecular level, the specific mechanism has not been fully elucidated. RB family proteins (including RB, p130, and p107) are involved in cell cycle regulation, a process that largely depends on members of the *E2F* gene family that encode transcriptional activators and repressors. In gastric cancer, inactivation of the RB-E2F pathway serves as a core transcriptional mechanism that drives cell cycle progression, and is regulated by cyclins, cyclin-dependent kinases, cyclin-dependent kinase inhibitors, p53, *Helicobacter pylori* and some other upstream molecules. The E2F proteins are encoded by eight genes (i.e. *E2F1* to *E2F8*), each of which may play a specific role in gastric cancer. Interestingly, a single E2F such as E2F1 can activate or repress transcription, and enhance or inhibit cell proliferation, depending on the cell environment. Thus, the function of the E2F transcription factor family is very complex and needs further exploration. Importantly, the presence of *H. pylori* in stomach mucosa may affect the RB and p53 tumor suppressor systems, thereby promoting the occurrence of gastric cancer. This review aims to summarize recent research progress on important roles of the complex RB-E2F signaling network in the development and effective treatment of gastric cancer.

## Introduction

Gastric cancer (GC) is a common type of gastrointestinal cancers. Worldwide it is the fifth most frequently diagnosed cancer and the third leading cause of cancer death (1). Although activation of proto-oncogenes and inactivation of tumor suppressor genes are considered as driving forces for GC, the pathogenesis of GC is a complex biological process mediated by both abnormal regulation of multiple genes and environmental insults (2). In recent years, the incidence of GC in western countries has been reduced, but it is still a serious public health problem in developing countries (1). Risk factors include *Helicobacter pylori* (*H. pylori*) infection, pickled food, smoking, obesity, chronic gastritis, and iron deficiency (3, 4). The most commonly used classification of GC is the two-category classification based on Lauren’s criteria: intestinal type and diffuse type, which are different not only morphologically, but also clinically and epidemiologically (5). The intestinal type is highly differentiated with a distinct premalignant state during cancer development, whereas the diffuse type is poorly differentiated lacking obvious premalignant lesions (5).

It is well known that tumorigenesis is a complex biological process usually mediated by polygenic mutations. The retinoblastoma (*RB*) gene (i.e. *RB1*) is the first tumor suppressor gene cloned in humans by positional cloning (6). It plays an important role in cell cycle regulation by regulating the adenoviral early region 2 binding factor (E2F) transcription factor family (7–10). The RB-E2F pathway not only regulates the cell cycle, but is also regulated by the cell cycle (10). In essence, it links the cell cycle to the transcriptional machinery, and plays a major role in the control of cell growth, apoptosis and differentiation, biological processes that are implicated in cancer development (9, 11).

The role of RB family proteins in GC was last reviewed in 2010 (12). Although much progress has since been made in understanding how the RB-E2F pathway is involved in the pathogenesis of GC, the specific role of E2F family members and the RB-E2F pathway in GC has not been systematically reviewed since a review article on the role of E2Fs in cancers of digestive system was published in 2013 (13). In this review, we will discuss research progress on the role of RB and E2F family members as well as their major upstream regulators in the initiation, progression and prognosis of GC. In addition, we will also summarize major research findings on how *H. pylori* infection impacts the development of GC by functionally disrupting the RB and p53 tumor suppressor systems. Finally, we will discuss major clinical implications of this research progress in effective treatment of GC.

## Gastric Cancer

Intestinal gastric cancer (IGC) is thought to be initiated primarily by *H. pylori* infection, with higher incidence in older men in high-risk areas (14, 15). Well differentiated and poorly differentiated gastric adenocarcinomas usually harbor different genetic changes, with well-differentiated being more frequently associated with changes in important cancer-related genes such as *RB* and *PTEN* (16). IGC has a relatively clear development process that is called metaplasia-neoplasia-carcinoma sequence or Correa’s cascade, from atrophic gastritis to intestinal metaplasia (IM) to dysplasia and then to IGC (17). IM is a recognized premalignant lesion of gastric mucosa, defined as the replacement of gastric mucosa by epithelial cells with intestinal morphology, and is associated with an increased risk of GC (18, 19). IM can be either complete (with the large-intestine phenotype) or incomplete (with the small-intestine phenotype), with the latter more frequently associated with malignant transformation (18). In a 10-year prospective study published in 2018, it was found that IM cells had both genetic and epigenetic mutations that differed from GC cells (20). For example, *TP53* and *ARID1A*, which are involved in the regulation of the RB-E2F pathway, are the most frequently mutated genes in GC, but are rarely mutated in IM (20). However, the exact mechanism of different genomic and epigenetic alterations between IM and GC and their application value in the prevention of GC still need to be explored (20).

Diffuse gastric cancer (DGC) usually results from pangastritis, has no atrophy and occurs mainly in younger female patients in low-risk areas (14, 15). DGC is poorly differentiated with stronger metastasis and invasiveness, and is often associated with CDH1 deficiency (21). By exploring the co-expression network of GC-related genes, an integrative functional genomics study group revealed differences between the two major subtypes of GC in transcriptional and epigenetic regulations as well as in stem cell characteristics (22). IGC was believed to be more affected by E2F-mediated transcription (22). Considering different characteristics of the two subtypes, development of subtype-specific targeted treatment strategies for GC deserves more attention.

**Figure 1:**
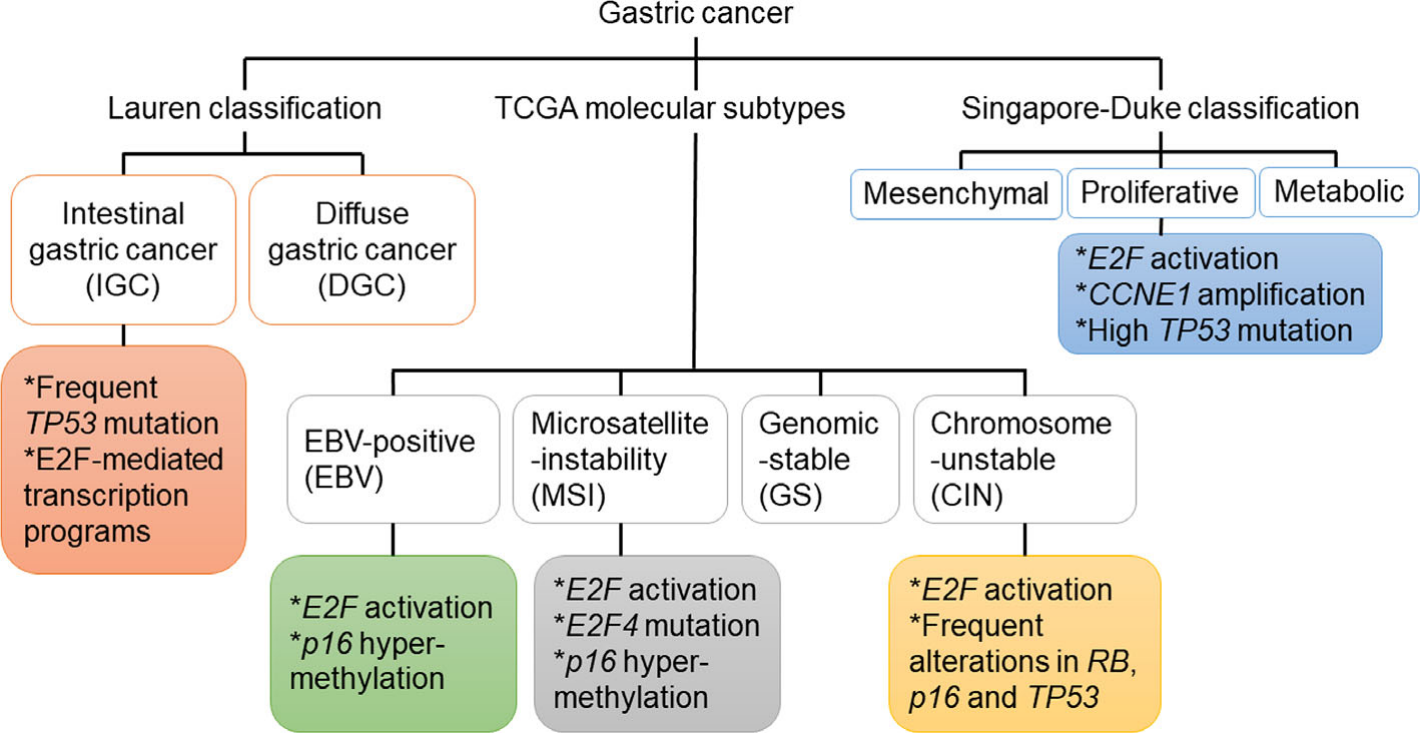
Genetic and epigenetic mutations of genes in key components of the RB-E2F pathway that were identified in different subtypes of gastric cancer.

In addition to the aforementioned Lauren’s classification, GC can also be divided into four molecular subtypes based on analyzing the data from The Cancer Genome Atlas (TCGA) project (23). The first subtype is Epstein–Barr virus (EBV)-associated GC, accounting for about 10% of GC (24). The association between EBV and GC was first recognized in 1990 (25). EBV has been shown to induce the nuclear export of E2F4 and E2F5 to prevent cell cycle arrest, an action that may have implications for the pathogenesis of GC (26). The second subtype is microsatellite unstable GC, accounting for 15–20% of GC. The hallmark of this subtype is microsatellite instability (MSI), accompanied with increased gene mutation rates (23). The intracranial histological heterogeneity of GC with MSI was associated with progressive frameshift mutations of TGF- receptor type II and *E2F-4* (27). High levels of MSI were more common in IGC and in the antrum, with better differentiation and more lymphoid infiltration (28). The other two molecular subtypes of GC are genomic stable (GS) GC and GC with chromosomal instability (CIN) (23), which includes poorly differentiated endocrine carcinomas that are often accompanied with the inactivation of p53- and RB-related pathways (29). Interestingly, a similar study based on gene expression profiling identified three subtypes of gastric adenocarcinoma: proliferative, metabolic, and mesenchymal, with the proliferative subtype being often associated with the activation of E2F-mediated pathway (30). Since patients with different subtypes likely have different clinical characteristics and molecular basis, they may benefit from different treatments. Abnormalities of key components in the RB-E2F pathway identified in patients with GC are summarized in **Figure 1** and **Table 1**.

**Table 1:** Abnormalities in key components of the RB-E2F pathway in patients with GC.

Genes	Alteration prevalence (%)						
	mRNA high	mRNA low	Loss of protein	Positive immunostaining	Methylation	Mutation	Amplification
E2F1	40 (31)	(−)	(−)	22.2 (32), 63 (31)	(−)	(−)	4 (31)
E2F3	(−)	70 (31)	(−)	(−)	(−)	(−)	(−)
E2F4	(−)	(−)	(−)	(−)	(−)	31–33 (33, 34)	(−)
E2F6	(−)	(−)	(−)	46 (35)	(−)	(−)	(−)
RB	(−)	(−)	33–40 (36, 37)	53–70.2 (38–39–42)	17.9 (43)	(−)	(−)
p130	(−)	(−)	(−)	nucleus: 25 (44)	(−)	(−)	(−)
				cytoplasm: 76.05 (44)			
cyclin D1	40.5 (45)	(−)	(−)	37–72 (36–38, 40, 45, 46)	(−)	(−)	16.6 (45)
CDK4	(−)	(−)	(−)	61.9 (40)	(−)	(−)	(−)
p16	(−)	(−)	22 (47), 49 (36, 37)	27.5–58.3 (40, 41, 48, 49)	72.6 (43)	(−)	(−)
p14^ARF^	(−)	(−)	(−)	45.2 (40)	24 (50)	(−)	(−)
p53	(−)	(−)	(−)	39–64 (39, 42, 49)	(−)	44.4 (51)	(−)

(-) represents no published data.

## The RB Family

The RB family consists of three members in humans, which are collectively referred to as “pocket proteins” and are involved in the regulation of the cell cycle (52). They are also involved in many biological processes such as proliferation, differentiation, senescence, apoptosis, gene regulation, and interact with many other cellular proteins (53, 54). The eponymous member of the pocket protein gene family is *RB1* or *RB*, which was named from an inherited eye tumor called retinoblastoma (55). The *RB* gene was mapped on chromosome 13q14.2 (6). RB is widely distributed in various tissues and interacts with a large number of transcription factors and chromatin-remodeling proteins, allowing itself to bind to transcription factors and to modify chromatin structure (56). In addition to regulating the cell cycle, RB has also been shown to inhibit apoptosis (57). Consistent with an important role of RB in tumorigenesis, loss of function of RB has been associated with the development of many human cancers (58–64).

The second member of the family is *p130*, which was cloned in 1993 and mapped on chromosome 16q12.2 (65, 66). The third member of the family is *p107*, which was mapped on 20q11.2 (67). Interestingly, the three pocket proteins have overlapping and interdependent functions (68). In both quiescent and *p53* activation conditions, RB and p130 can cooperate to repress G1/S genes, a process that RB plays a predominant role (69, 70). In the absence of RB and p130, p107 can also repress G1/S genes (69). Under the condition of DNA damage, p130 and p107 can cooperate to repress the G2/M genes and thus block cell cycle entry into mitosis (69). In general, when DNA damage leads to *p53* activation, RB, p130 and p107 cooperatively repress G1/S genes while p130 and p107 cooperatively repress G2/M genes (69). In mice, pocket proteins have overlapping functions in suppressing the development of various types of tumors. For example, RB and p107 worked together to suppress the development of retinoblastoma (71, 72), head and neck cancers (73), and spontaneous skin tumors (74). In addition, RB and p130 worked together to suppress the development of retinoblastoma (75, 76).

## The E2F Family

The E2F family of transcription factors includes 10 members, encoded by eight different genes, *E2F1–E2F8* (9). *E2F3* consists of two isoforms, *E2F3a* and *E2F3b*, derived from two different promoters (77). Members of the E2F family have both distinct and overlapping functions, and are important for various biological processes such as cell cycle control, cellular proliferation and apoptosis (78, 79). E2F1–6 are canonical E2Fs, which form heterodimers with dimerization partner (DP) proteins (80). E2F7 and E2F8 are atypical E2Fs, which do not bind to DP but have two DNA binding domains (9). All E2F members can bind to DNA in a sequence-specific manner to initiate transcriptional activation or repression of target genes (80). E2F1-3a are transcriptional activators, whereas E2F3b-8 are transcriptional repressors (9). However, it is worth noting that E2F3b can also act as a transcriptional activator (81, 82), even though its expression pattern during the cell cycle is similar to that of a canonical E2F repressor (77). The functional specificity of E2F-DP complex is determined by the E2F subunit, but in the absence of DP, E2Fs become non-functional (78).

In quiescent cells, pocket proteins can bind to E2F-DP heterodimers to repress E2F target genes. It is worth noting that different pocket proteins preferentially bind to different E2F transcription factors (83). RB binds to E2F1, E2F2, and E2F3 to form the repressive RB-E2F complex, while p107 and p130 bind to E2F4 and E2F5 to form the repressive DREAM (DP, RB-like, E2F and MuvB) complex (10). E2F6, E2F7 and E2F8 are not bound by pocket proteins (9). In G0 and early G1 phases, hypophosphorylated RB, which is in an activated state, binds to the pocket domain of E2F1-3 and inhibits E2F-mediated target gene activation, thereby blocking cell cycle progression at the G1/S transition (84). In addition, E2F4 and E2F5 can form complexes with p107 and p130 to mediate gene repression (84). When cells receive growth stimuli, activation of cyclin dependent kinases (CDKs) leads to the phosphorylation of pocket proteins and collapse of the previously formed RB-E2F complexes and DREAM complexes (70). The subsequent release of E2F1-3 from those complexes can activate target genes required for cell cycle entry (10, 52).

## Pocket Proteins in Gastric Cancer

Various studies showed that RB plays important roles in the various aspects of GC. However, earlier studies focused on evaluations of its protein levels in various contexts of GC appeared to yield seemingly conflicting results. For example, compared with non-neoplastic tissues, tumors could have higher (38) or lower (48) levels of RB. In addition, altered RB protein levels were more frequent in less-invasive GC than in advanced invasive GC (85). In univariate and multivariate analyses, positive *RB* expression was found to be significantly correlated with the presence of lymph node metastasis (39). Nevertheless, another study showed that the expression of *RB* in lymph node metastasis was lower than that of the corresponding primary tumor (36). These inconsistent data may be related to the fact that RB function is largely dependent on its posttranslational regulation (i.e. phosphorylation). Therefore, defining the precise role of RB in various processes of GC likely requires evaluation of its phosphorylation status instead of just its mRNA or protein levels. In addition, since RB function is manifested at least in part through limiting activities of activator E2Fs, evaluating the RB status in GC patient samples will benefit from simultaneously evaluating the status of activator E2Fs. It is interesting to note that DNA methylation of the *RB* gene promoter was found in significantly more GC samples (17.9%) than in normal samples (5.5%) (43), suggesting that *RB* methylation may also play a role in GC.

Although little is known about the precise role of p107 in GC, cellular localization of p130 seems to play an important role in some aspects of GC. For example, high levels of nuclear localization of p130 were significantly correlated with lower grade GC, whereas high levels of cytoplasmic localization of p130 were significantly correlated with IGC (49). Besides, p130 was localized in the cytoplasm in DGC but in the nucleus in normal cells, further supporting an important role of its nuclear delocalization in the development of GC (44). However, no correlation has been found between cytoplasmic localization of p130 and tumor grade or survival of DGC. Although the functional consequence of p130 nuclear delocalization on the development of GC is currently unclear, it is plausible that such delocalization promotes the development of GC through inhibiting the function of p130 as a transcriptional modulator. Further investigations are needed to experimentally determine the precise role of p130 nuclear delocalization in GC and its underlying mechanisms.

Since the 2010 review, two significant advances have been made in the understanding of pocket proteins in GC. The first interesting and important finding was that p130 was primarily localized in the nucleus in normal cells but was mainly localized in the cytoplasm in DGC cells (44). Future studies should be directed to understanding the precise role of p130 subcellular localization in GC, the nucleo-cytoplasmic shuttling mechanisms, and whether p130 nuclear delocalization facilitates GC development by impairing p130-mediated transcriptional repression. In addition, it has been found that besides RB phosphorylation, *RB* promoter methylation may also play a role in the development of GC (43), highlighting the importance of epigenetic regulation of pocket proteins in GC. It would be interesting to know whether *RB* promoter methylation levels are different among various subtypes of GC, or among different stages of GC development.

## The E2F Family in Gastric Cancer

Among E2F family members, E2F1 is so far the most widely studied in tumors, including GC. It is interesting to note that *in vitro* different levels of E2F1 had different effects on cell fate: low levels of E2F1 could promote cell cycle progression, medium levels of E2F1 could cause cell cycle arrest, and high levels of E2F1 could lead to cell apoptosis (86). Several earlier studies using either transgenic mouse models or *in vitro* systems showed that the role of E2F1 in tumorigenesis was pleiotropic, manifested by the fact that it might either promote or suppress tumorigenesis, depending on dominant signaling pathways and cell types (87–91). In GC, *E2F1* gene amplification was rare, but its overexpression was detected in about 40% of patients (31). Gene expression microarray data and bioinformatic analysis of public datasets also showed that E2F1 was up-regulated in GC (92, 93). In addition, high mRNA levels of *E2F1* were related to poor survival (93). In order to better understand the role of E2F1 in biological processes of GC, various research groups investigated effects of *E2F1* overexpression or knockdown on the tumorigenicity of GC cells. For instance, overexpression of *E2F1* in MGC-803 GC cell line led to significantly increased levels of apoptosis but significantly reduced levels of cellular proliferation and invasiveness, consistent with the tumor suppressor function of E2F1 (94). In addition, overexpression of *E2F1* suppressed tumor growth and promoted tumor cell apoptosis in nude mice implanted with *E2F1*-overexpressing MGC-803 cells (95). Furthermore, adenovirus-mediated overexpression of *E2F1* in AGS and SNU-1 GC cell lines induced apoptosis and reduced cell survival rate (96). On the other hand, *E2F1* downregulation by intratumor-injection of *E2F1* shRNA in nude mice engrafted with MGC-803 cells inhibited tumor growth and promoted apoptosis, accompanied by up-regulation of *PTEN*, *Caspase-3* and *Caspase-9* (97). In addition, in cisplatin-resistant SGC7901/DDP cells, shRNA-mediated *E2F1* downregulation blocked cell cycle progression, promoted apoptosis, and increased the sensitivity of cells to several chemotherapeutic drugs, suggesting that *E2F1* served as an oncogene and promoted multidrug resistance in GC (98). Although the dual role of E2F1 in GC is likely context dependent, mechanisms underlying the seemingly inconsistent results are provided from the existing literature. For example, E2F1 is considered as a proto-oncogene, and elevated E2F1 levels are sufficient to drive cell proliferation and cell cycle progression (99, 100), which can also explain *E2F1* overexpression corresponding to poor prognosis (101). On the other hand, the tumor suppressive effect of E2F1 can be explained by E2F1-mediated apoptosis and growth arrest (102–104). E2F1 can inhibit the degradation of p53 by inducing the expression of p14^ARF^, leading to increased apoptosis and cell cycle arrest (105). In addition, E2F1 can also induce p53 independent of p14^ARF^ (103). This also explains why RB-negative tumors tend to be p53 negative, probably to avoid the negative E2F1-p14^ARF^ feedback (106). Interestingly, E2F1 protein levels could also reflect the sensitivity of GC patients to adjuvant chemoradiotherapy after radical gastrectomy. For example, among postoperative patients receiving adjuvant chemoradiotherapy, the E2F1 immuno-positive group had a higher survival rate than the E2F1 immuno-negative group (32). The immunopositivity of E2F1 might be used as an indicator for good response for adjuvant chemoradiotherapy and radiotherapy after surgery (107). A key determinant of the efficacy of anticancer therapies is the ability of cancer cells to undergo apoptosis in response to DNA damage factors (108). The success of radiotherapy or chemotherapy is at least partly due to the fact that cancer cells are more likely than normal cells to die when induced by DNA damage. Under the condition of DNA damage, E2F1 induces apoptosis through activation of various cell death pathways, which may explain the higher sensitivity of samples with high E2F expression to radiotherapy and chemotherapy (109).

Given the important role of E2F1 in GC, E2F1 has been considered as a potential therapeutic target for GC patients (93). However, since E2F1 activity is also important for normal cellular proliferation, therapeutically targeting E2F1 may have significant side effects on normal tissues that are capable of proliferating. In addition, due to the highly overlapping and compensatory effects of E2F activators (78), simple targeted intervention of E2F1 may lead to compensational upregulation of other two E2F activators, making such a therapy less effective. Therefore, E2F1 targeted therapy may require simultaneously targeting the other two E2F activators to achieve a better clinical outcome. Furthermore, the bidirectional effect of E2F1 on GC suggests that success on the targeted therapy is likely dependent on a clear understanding of the predominant oncogenic pathways involved in individual patients.

There are also several studies on E2F4 in GC. *E2F4* mutation was found to be a common and an early event in the occurrence of GC, and might occur in the process of precancerous lesions such as IM and dysplasia (33, 34). *E2F4* mutation in gastrointestinal tumors might not be random as it appeared frequently in a microsatellite region at exon 7 with a serine-encoding trinucleotide repeat sequence (33, 110). In addition, *E2F4* frameshift mutation was associated with differentiation grades of GC as frameshift mutation of the microsatellite regions encoding serine repeats might inhibit the formation of RB-E2F4 complex and reduce the level of differentiation (27). Furthermore, a study of MSI suggested that *E2F4* might be involved in the transformation of gastric adenocarcinoma into squamous cell carcinoma (111). Interestingly, by establishing an E2F-related transcriptional regulatory network, a research group found that target genes regulated by E2F1 and E2F4 showed a large number of differential expressions in GC, indicating that E2F1 and E2F4 might play important roles in tumorigenesis of GC (92). It was found that *E2F4* mRNA levels increased with the degree of tumor invasion and malignancy (92). Bioinformatic analysis of a Gene Expression Profiling Interactive Analysis (GEPIA2) dataset representing 408 GC samples and 211 normal tissues showed that there was no difference in average expression levels of *E2F4* between GC samples and normal tissues, but bioinformatics analysis using a completely different and consolidated Gene Expression Omnibus (GEO) dataset representing a much larger sample size (i.e. up to 876 GC samples) showed that patients with relatively high *E2F4* expression had worse survival than those with relatively low *E2F4* expression (93). As a member of the DREAM complex, E2F4 can repress many cell cycle genes (112), which are common markers of proliferation that can stratify most cancers, including GC (101). Although both high expression of *E2F4* in advanced GC and its correlation with poor prognosis are seemingly contradictory to the repressive role of E2F4 in cell cycle control, there are existing studies supporting an oncogenic role of E2F4. For example, E2F4 induced proliferation and promoted the development of skin tumors in a keratin 5 promoter-driven E2F4 transgenic mice (113). In addition, E2F4 reduced apoptosis in cardiac myocytes (114). However, more investigations should be done to explore the precise role and underlying cellular and molecular mechanisms of E2F4 in GC initiation and progression, and to determine whether *E2F4* overexpression is associated with a specific subtype of GC.

Compared to E2F1 and E2F4, other E2F family members have been much less studied regarding their potential roles in GC. Gene expression microarray data showed that mRNA levels of *E2F2* in GC samples were increased compared with those in normal samples (92). Using Northern blot technique to analyze 30 GC samples and their corresponding non-neoplastic mucosa, a Japanese research group found that mRNA levels of *E2F3* were lower in 70% of GC samples than in normal controls (31). In contrast, bioinformatic analysis of RNA sequencing data from a GEPIA2 dataset representing a much larger sample size (i.e. 408 GC samples and 211 normal gastric tissues) showed that expression levels of *E2F3*, along with *E2F2*, *E2F5*, *E2F7* and *E2F8*, were significantly higher in GC samples than those in normal tissues (93). These conflicting results on *E2F3* expression levels from the two studies may be due to differences in patients’ genetic background (i.e. mostly Japanese *vs.* mostly Caucasians and African Americans) and/or techniques (i.e. Northern blot *vs.* RNA sequencing) used to evaluate *E2F3* expression levels. Moreover, high levels of *E2F2*, *E2F3*, *E2F5*, *E2F6*, *E2F7* and *E2F8* were related to better survivals (93). E2F6 was localized in the nucleus, and was at high levels in gastric adenocarcinoma without lymph node metastasis (35). Similarly, univariate analysis showed that the expression of *E2F6* was negatively correlated with lymph node metastasis, suggesting that E2F6 might suppress the metastasis of GC (35). Thus it is clear that considerably more study is warranted to investigate the role and mechanism of E2F2, E2F3, E2F5, E2F6, E2F7 and E2F8 in GC. For example, it would be interesting to know whether all or some of the aforementioned upregulated E2F factors (93) are coordinately overexpressed in GC samples.

Since the summary of the role of E2F transcription factors in digestive tract malignancies in 2013, much progress has been made in understanding the roles of E2F family members in GC. There have been more data to explain the bidirectional effect of E2F1 on GC. In addition, the relationship between E2F1 and better chemoradiotherapeutic response in GC has been established. It is worth pointing out that the bidirectional effect of E2F1 and its effect on chemoradiotherapeutic sensitivity have also been found in many other tumors (109, 115). Furthermore, the application of bioinformatics has facilitated our understanding of GC-specific genetic alterations in various E2F members as well as their prognostic and other clinical implications.

## Upstream Regulators of the RB-E2F Pathway in Gastric Cancer

Many upstream regulators of the RB-E2F pathway also play important roles in GC. The activities of RB and other pocket proteins are mainly regulated by phosphorylation through CDKs, which are in turn regulated by cyclins and cyclin-dependent kinase inhibitors (CKIs) (116, 117). Therefore, cyclins, CDKs, and CKIs as well as any molecules that regulate these three types of proteins may be involved in the pathogenesis of GC.

The cyclin D1 protein was almost undetectable in normal gastric mucosa, but was elevated in about half of GC cases, indicating that overexpression of *cyclin D1* might be an early event in the process of tumorigenesis in GC (45, 46). The *p16* gene, also known as *p16^INK4a^*, is located on chromosome 9p21 (118) and encodes for a protein that is an inhibitor of CDK4 (119, 120). As a CKI, p16 is able to competitively block the cyclin D1-CDK4 complex by binding to CDK4, an action that inhibits CDK4-mediated RB phosphorylation and prevents cell cycle progression from G1 to S phase (118). Loss of p16 function leads to an abnormal increase in cyclin D1-CDK4 complex activity, resulting in sustained RB phosphorylation (118). At the same time, phosphorylation of RB in G1 phase results in increased expression of *p16* to limit CDK4 activity (118). This negative feedback loop of p16 and RB is critical for normal cell cycle control to protect cells from abnormal cellular proliferation. Therefore, deregulation of key components in the feedback loop is likely associated with the development of GC. For example, various p16 abnormalities have been identified in GC patient samples. An early study showed that about 50% of GC samples were detected with the loss of *p16* expression (36). Interestingly, the expression of *p16* in distal gastric carcinomas was higher than that in gastric cardia carcinomas (40). In GC, abnormal methylation of CpG islands in the promoter region of *p16* downregulated *p16* (47). Methylation of *p16* was present in about 70% of GC samples, while there was almost no *p16* methylation in normal samples (43). In addition, methylation of *p16* was found in both IGC and DGC, but had no significant correlation with either tumor staging or histology (50). It is worth noting that hypermethylation of *p16* significantly increased in MSI-high GC (121). Furthermore, *p16* hypermethylation is also very common in EBV-associated GC, and may even be one of the important causes of EBV-associated GC (122, 123). The expression levels of *p16* and *RB* were not only altered in GC, but also negatively correlated (41, 124, 125).

**Figure 2:**
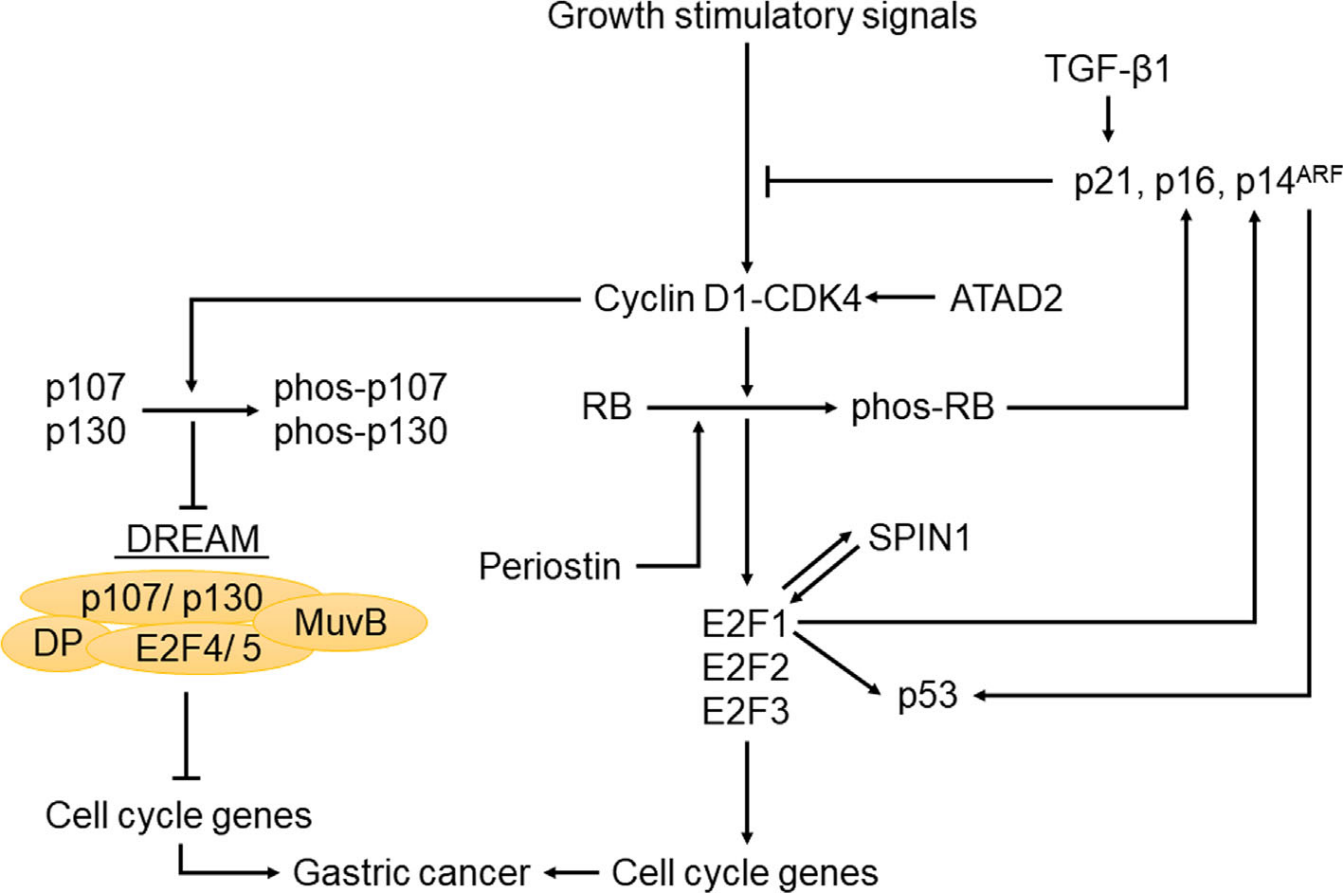
The regulatory network of the RB-E2F pathway in gastric cancer. When cells receive growth stimuli, activation of CDKs leads to the phosphorylation of pocket proteins, collapse of the previously formed RB-E2F complexes and DREAM complexes. The subsequent release of E2F1-3 can activate target genes required for cell cycle entry. Meanwhile, this pathway is also regulated by upstream and downstream molecules in gastric cancer, such as CKIs, p53, TGF-β1, ATAD2 and SPIN1.

Several other upstream regulators in the RB-E2F pathway have also been implicated in GC. For example, transforming growth factor-beta1 (TGF-β1) inhibited GC cell growth by upregulating its downstream target p21, thereby blocking p130 phosphorylation and preventing aberrant cell cycle progression by downmodulating CDK activities (126). In addition, the tumor suppressor function of periostin was achieved by its induction of RB phosphorylation and the subsequent release of E2F1, which activated its target gene *p14^ARF^*, leading to the inactivation of *MDM2* and the consequential reduced ubiquitination of p53 and E-cadherin (127). Moreover, SPIN1 could form a positive feedback loop with E2F1 to promote the development of GC (128). Furthermore, *ATAD2* knockdown in GC cells led to reduced levels of cyclin D1, cyclin E, E2F1 and RB phosphorylation, thus inhibiting proliferation and cell cycle progression (129). Interestingly, decrease of intracellular chloride ion concentration could increase the level of p21 and reduce the phosphorylation of CDK2 and RB (130). This effect led to cell cycle arrest and inhibited the growth of tumor cells, providing us with a new therapeutic strategy (130). The fact that many upstream molecules of the RB-E2F pathway have a large proportion of genetic and epigenetic alterations in GC (**Figure 1** and **Table 1**) suggests that in addition to the downstream effectors of RB such as activator E2Fs, its upstream regulators such as p16 and cyclin D1 also play important roles in GC, with the specific regulation network shown in **Figure 2**. Therefore, understanding the status of the upstream regulators of RB will not only further help us better understand the functional role of RB in various process of GC, but may also provide additional insights on the diagnosis, prognosis and effective treatment of GC.

## *Helicobacter pylori* and the RB and p53 Tumor Suppressor Systems

The *p53* tumor suppressor gene, also known as *TP53*, was first discovered in 1979 (131). In cells under non-stressed condition, p53 is usually present in small amounts (132). However, in the case of stress, such as hypoxia, DNA damage, proto-oncogene activation, radiotherapy and chemotherapy, p53 protein is stabilized to initiate a damage response cascade (132). If the damage cannot be repaired in time, p53 would induce apoptosis by binding to the apoptosis stimulating proteins of p53 (ASPP) (133, 134). Like RB, the p53 tumor suppressor also controls cell cycle but through independent and interrelated pathways (135). Therefore, it is not surprising that alterations of *TP53* and *RB* are common events in human GC. It was reported that *TP53* gene mutations were found in about 50% of GC cases (51).

*H. pylori* is a Gram-negative bacterium that was found in stomach mucosa, and is an important risk factor for GC, equivalent to a type I carcinogen (136). About half of the population in this world has *H. pylori* infection, and infection rates in Asian countries are generally higher than those in western countries (137). *H. pylori* could cause abnormal DNA methylation and inflammation, which increased the risk of GC (138). However, there was no significant difference in the rates of *H. pylori* infection either between IGC and DGC, or between proximal and distal tumors (139). Interestingly, high levels of *RB* methylation in *H. pylori*-positive individuals might increase the risk of GC (140). The proportions of RB tumor suppressor and the p53 tumor suppressor pathway abnormalities in *H. pylor*i-infected GC were higher than that in non-*H. pylori*-infected GC (42). It was reported that *H. pylori* infection might first activate *C-MYC* and *BCL-2* in IM, and then inactivate the RB and p53 tumor suppressor pathways in dysplasia, causing a severe imbalance of proliferation and apoptosis in precancerous lesions, leading to the occurrence of GC (42).

The pathogenicity of *H. pylori* is mainly due to its flagellum, lipopolysaccharide, vacuolar toxin VacA, and cytotoxin-related gene pathogenicity island (cagPAI) (141–143). VacA could generate a protective intracellular reservoir where *H. pylori* survives by usurping lysosomal and autophagy pathways. Besides, it was found that gastric epithelial cell apoptosis induced by VacA did not require RB regulation, and occurred whether or not p53 was expressed (144). The most important and widely studied virulence factor of *H. pylori* strains is cytotoxin-associated protein (CagA). CagA is introduced into gastric epithelial cells by the type IV secretion system (T4SS), leading to the promotion of genetic instability, epithelial–mesenchymal transformation and eventual carcinogenesis (145). In addition, CagA bound to ASPP2, thereby inhibiting the binding of ASPP2 to p53, leading to decreased apoptosis and promoting the formation of GC (146, 147).

*H. pylori* could activate the PI3K/AKT pathway (148, 149) or the MAPK/ERK pathway (150) to activate the ubiquitin ligases murine double minute (MDM2, also known as HDM2), which promoted ubiquitination and proteasomal degradation of p53. On the other hand, p53 can activate MDM2 to form a negative feedback loop that ensures low levels of p53 in unstressed cells (151). Related proteins of the p53-MDM2 feedback loop were distinctly expressed at different stages of GC development (152). In the case of *H. pylori* infection, *MDM2* expression was found to be significantly elevated in the progression from chronic gastritis to GC (152). Interestingly, MDM2 was bound to RB through a central acidic domain in U20S, C33A, SAOS-2 cells (153), and could promote proteasomal degradation of RB in cells of osteosarcoma, cervical cancer, non-small cell lung cancer and temperature-sensitive murine ts20 cells (153, 154).

**Figure 3:**
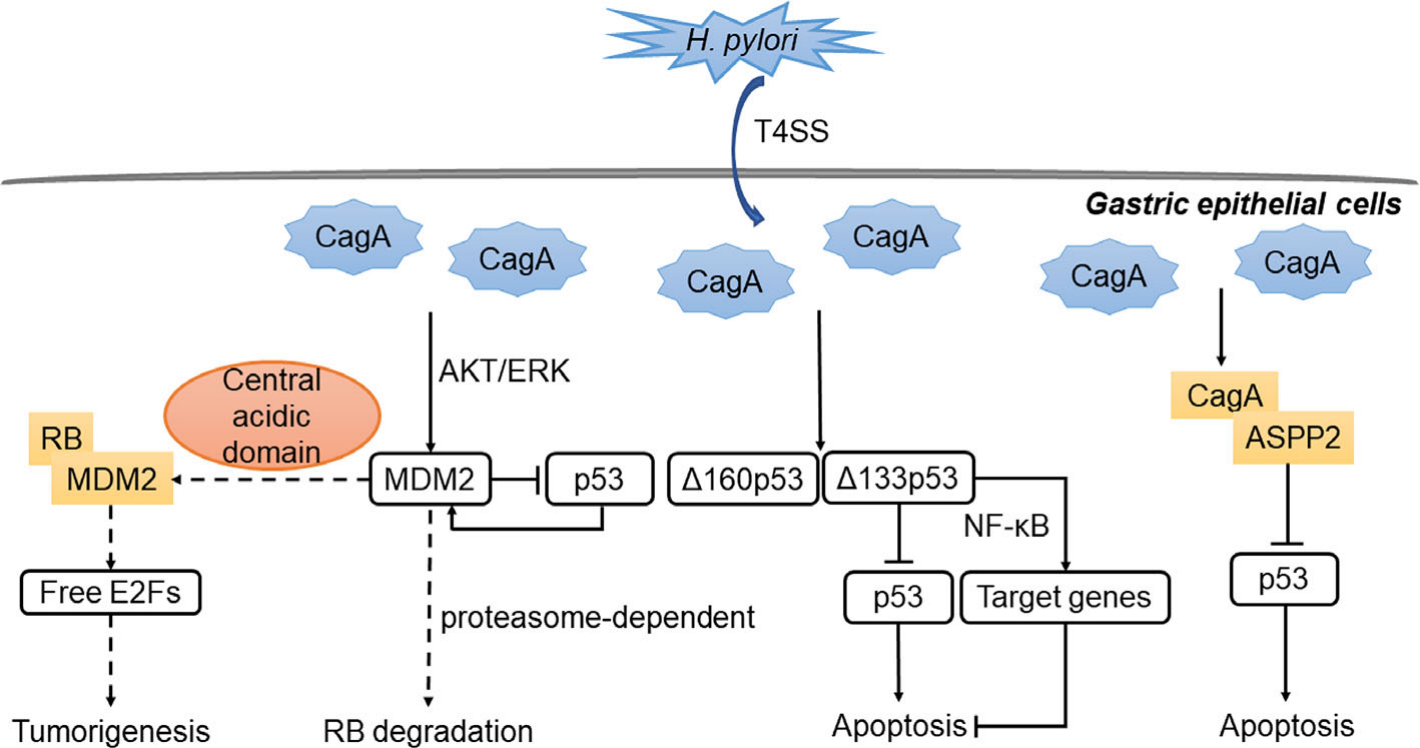
Regulation of p53 and RB by *H. pylori* in stomach. The virulence factor CagA is introduced into gastric epithelial cells by T4SS. CagA binds to ASPP2, thereby inhibiting the binding of ASPP2 to p53, leading to decreased apoptosis. Besides, MDM2 is activated through the AKT/ERK pathway and form a negative feedback loop with p53. MDM2 can bind to RB and inhibits the function of RB-E2F repressor. Meanwhile, the expression of the isoforms of p53 inhibits the activity of p53 or activates NF-κB target genes. The outcome is the down-regulation of apoptosis and the occurrence of gastric cancer.

*H. pylori* could also induce a subtype-specific damage response mechanism of p53 in a T4SS-dependent manner (155). Specifically, *H. pylori* induced Δ133p53 and Δ160p53, which encode for N-terminally truncated isoforms of p53 protein, thereby inhibiting the activity of p53. Δ133p53 also activated the NF-κB pathway and caused up-regulation of its downstream target genes, leading to the inhibition of apoptosis indirectly (155). There is also cross talk between p53 and NF-κB pathway that results in reduced apoptosis and the occurrence of tumor (156). Besides, some *H. pylori* products were associated with the RB-E2F pathway *in vitro* (157, 158). By isolating and cloning genes encoding for the two secretory *H. pylori* proteins CagA and HspB, and transfecting them into AGS cell line, researchers found that CagA and HspB directly promoted the growth of GC cells by facilitating G1-S transition of the cell cycle through the upregulation of *cyclin D3* and subsequent RB phosphorylation (158). Interestingly, unknown soluble factor(s) released by *H. pylori* in cell culture medium might inhibit RB phosphorylation by increasing the level of p27, leading to inhibition of cell cycle progression in gastric epithelial cells (157). The primary interactions between *H. pylori* and the RB and p53 tumor suppressor pathways are summarized in **Figure 3**.

Studies based on clinical practice have shown that treatment of *H. pylori* reduced the risk of precancerous lesions converting to GC, but the degree of risk reduction depended on the population and the extent of damage already present at the time of eradication (159–161). The close association between *H. pylori* and the RB and p53 suppressor pathways provides us with the possibility of combinational therapy, such as *H. pylori* eradication combined with targeted intervention of MDM2 or other related molecules, which may greatly improve the therapeutic effect. Interestingly, the positive index of E2F nuclear staining was higher in *H. pylori*-infected gastric mucosa than in non-infected gastritis samples, and E2F1 was co-localized with proliferating cell nuclear antigen (PCNA) (162). The positive index of E2F1 decreased after *H. pylori* successful eradication (162). This study suggests that enhanced expression of E2F may be involved in the occurrence and development of *H. pylori*-infected GC by promoting cell cycle progression (162). Therefore, we speculate that E2F-targeted therapy may be more effective in patients with *H. pylori*-infected gastritis and *H. pylori*-infected GC, and has the potential to be applied in the prevention and treatment of GC.

## Potential Therapeutic Targets of the Rb-E2f Pathway in Gastric Cancer

Because GC is often asymptomatic in the early to middle stage of the disease progression, it is often diagnosed at an advanced stage with limited treatment options. Currently the primary treatment strategy for GC is still surgeries, complemented with chemotherapy and radiotherapy (163). Most patients still have low survival rates and high recurrence rates (163). Therefore, it is particularly important to find more effective treatment strategies and preventive measures for GC. Through studies on the function of tumor suppressor genes and mechanisms of related pathways such as the RB-E2F pathway, we may be able to find novel therapeutic targets and develop more effective treatment strategies for GC. Antagonists of CDK can block the action of the cyclin D1-CDK4 complex to target the RB-E2F pathway for cancer therapy (164). Flavopiridol is a broad-spectrum CKI commonly used in clinical practice of solid tumors (165). A phase II clinical trial showed that flavopiridol alone had no significant antitumor effect on advanced GC (166), pending changes in regimen and combination with other agents. Selective CDK4/6 inhibitors palbociclib, ribociclib and abemaciclib have been developed and are undergoing clinical trials in a variety of cancers (167). Palbociclib is in phase II trials in patients with advanced GC with limited single-agent activity (168). Some of the genetic characteristics of GC help us stratify patients for the most effective drug therapies. Studies showed that high levels of cyclin E protein in GC correspond to increased resistance to palbociclib (169). The methylation of *p16* increased the sensitivity of GC cells to abemaciclib, suggesting that abemaciclib is more effective in patients with hypermethylated *p16* (170). Targeted therapies with a single drug are likely to develop drug resistance, but combinations of drugs are more effective in controlling the disease. For example, palbociclib had a synergistic effect with 5-FU in the treatment of GC cells (169). Combination of human epidermal growth factor receptor 2 (HER2) inhibitor pyrotinib and CDK4/6 inhibitor SHR6390 was thought to be a more effective treatment strategy for HER2-positive metastatic GC (171). In **Table 2**, we summarize the CKIs currently used in clinical trials and preclinical studies for GC. Notably, CDK4/6 inhibitor relies on RB to induce cell growth arrest (172). In order to improve therapeutic efficacy and precision, we need to develop new therapeutic strategies, such as the use of multiple CDK4/6 inhibitors to enhance cell cycle arrest and selective targeting of *RB*-deficient tumors (172). Immunotherapy based on immune checkpoint block is being applied in the clinical treatment of advanced GC, such as anti-PD-1 therapy (163). However, most GC cases are not sensitive to immune checkpoint inhibitor monotherapy, so patients may need combinational therapies to improve response to the PD-1 therapy or other immune checkpoint inhibitors (163). If CDK4/6 inhibitors can be combined with immunotherapy in the treatment of GC in the future, perhaps a better therapeutic effect will be achieved.

**Table 2:** CKIs for clinical trials and preclinical studies in GC.

CKIs	Status	Subjects	Settings	Results
Flavopiridol (166)	Phase II clinical trial	16 advanced gastric carcinoma patients	single-agent administration	No anti-tumor activity unexpected side effect
Palbociclib (168)	Phase II clinical trial	29 advanced gastro-esophageal cancer patients	single-agent administration	Limited anti-tumor activity
Palbociclib (169)	Preclinical research	GC cell lines	cyclin E overexpression	Elevated resistance
Palbociclib (169)	Preclinical research	GC cell lines	Combined with 5-FU	Better anti-tumor effect
Abemaciclib (170)	Preclinical research	146 GC patients & GC cell lines	p16 hypermethylation	Elevated sensitivity
SHR6390 (171)	Preclinical research	GC cell lines & AVATAR mice	Combined with pyrotinib	Better anti-tumor effect
SHR6390 (171)	Phase I clinical trial (ongoing)	fives GC patients	Combined with pyrotinib	PR in three patients, SD in one patient, PD in one patient (until June 2020)

PR, partial response; SD, stable disease; PD, progressive disease.

Using an E2F promoter-regulated adenovirus carrying the *p16* gene could combine the apoptosis induced by *p16* gene and oncolysis induced by virus replication to have antitumor effect on GC (173). This kind of replication-competent adenovirus (RCAd) provided a new view of cancer therapies (173). At present, oncolytic virus has become an active research field on cancer targeted therapies (174). Advances in genetic engineering can help scientists create oncolytic viruses that target cancer cells with different types of mutations to achieve better therapeutic effects (174). If oncolytic virus and immunotherapy are properly combined in GC, it is possible to achieve a synergistic anti-cancer effect. However, since there are significant uncertainties on potential side effects and viral penetration efficiencies in solid tumors like GC, such a therapy option still has a long way to go before it can be used in clinical practice of GC.

*H. pylori* eradication therapy has been widely used in clinical practice and significantly reduced the risk of GC (160, 175). An experiment using *H. pylori*-infected *p27*-deficient mice showed that *H. pylori* eradication through an antibiotic combinational therapy could reduce gastric inflammation and hinder precancerous lesions such as gastric ulcer and dysplasia, thus preventing GC (176). Interestingly, *H. pylori* induced cytoplasmic localization of p27, resulting in loss of tumor suppressor function of p27 and correspondingly poor prognosis of patients (177). CDK4/6 inhibitors play the same role as p27, so patients with p27 cytoplasmic localization after *H. pylori* infection may respond better to CDK4/6 inhibitors. In addition, *H. pylori* activates many cell cycle-related genes, such as *E2F1* and *cyclin D1* (178), suggesting that current CDK4/6 inhibitors and potential RB-E2F targeting agents may be more effective in *H. pylori*-infected patients. *H. pylori* infection can also change the epigenetics of cells, such as increased *p16* methylation (179). In this regard, *H. pylori*-infected GC patients may be more sensitive to abemaciclib (170).

Intervention of RB-E2F pathway has not been commonly used in GC as virtually all such options remain in preclinical stages or in clinical trials. Nevertheless, targeted therapies based on key components of the RB-E2F pathway, combined with immunotherapy, oncolytic viral therapy, and/or *H. pylori* eradication are likely a viable option for developing more effective treatment strategies for GC.

## Conclusions and Perspective

In summary, RB-E2F pathway plays important roles in the occurrence and development of GC. Current understanding on the role and mechanism of the three pocket proteins in GC are insufficient, especially for p107. The functional consequences of epigenetic regulation of *RB* and cytoplasmic localization of p130, as well as the cooperative functions of these three pocket proteins in GC have great potentials to be explored. Most studies involving E2Fs in GC have focused on E2F1 and E2F4. We need a better understanding of the roles of other E2F family members in GC. In addition, whether E2F1 can be used as a viable therapeutic target remain to be determined. Since current data on the dual role of E2F1 in GC came from GC cell lines or xenograft mouse models, using transgenic mouse models will likely provide more significant insights on how E2F1 is involved in the various process of GC, including initiation, progression, and drug resistance. In view of the importance of *H. pylori* in GC and its complex interaction with the RB-E2F pathway, *H. pylori* eradication therapy combined with targeted therapy may achieve more effective therapeutic outcomes. It is worth noting that the molecular typing of GC is an important advance as we are now able to tailor our studies to different genetic alterations for each molecular subtype, thereby facilitating precision medicine. In the clinical application of RB-E2F pathway for GC, essentially all targeted therapy options remain in preclinical stages or in clinical trials. Therefore, there is an urgent need to facilitate our research efforts on translating research data into clinical practice. Nevertheless, targeting key components of the RB-E2F pathway for the development of more effective therapies of GC offers both significant promises and challenges to the scientific community.

## Author Contributions

Both authors (TW and LW) prepared, revised and edited this manuscript. All authors contributed to the article and approved the submitted version.

## Funding

This work was supported by the start-up funds from the China Medical University.

## Conflict of Interest

The authors declare that the research was conducted in the absence of any commercial or financial relationships that could be construed as a potential conflict of interest.
